# Hydrocephalus and Cerebrospinal Fluid Analysis Following Severe Traumatic Brain Injury: Evaluation of a Prospective Cohort

**DOI:** 10.3390/neurolint13040052

**Published:** 2021-10-19

**Authors:** Hansen Deng, Ezequiel Goldschmidt, Enyinna Nwachuku, John K. Yue, Federico Angriman, Zhishuo Wei, Nitin Agarwal, Ava M. Puccio, David O. Okonkwo

**Affiliations:** 1 Department of Neurological Surgery, University of Pittsburgh Medical Center, Pittsburgh, PA 15213, USA; nwachukuel@upmc.edu (E.N.); chris.wei@pitt.edu (Z.W.); agarwaln@upmc.edu (N.A.); puccioam@upmc.edu (A.M.P.); okonkwodo@upmc.edu (D.O.O.); 2 Department of Neurological Surgery, University of California San Francisco, San Francisco, CA 94143, USA; ezegold@gmail.com; 3 Department of Critical Care Medicine, Sunnybrook Health Sciences Center, University of Toronto, Toronto, ON M4N 3M5, Canada; john.yue@ucsf.edu (J.K.Y.); F.angriman@mail.utoronto.ca (F.A.); 4 Neurotrauma Clinical Trials Center, University of Pittsburgh Medical Center, Pittsburgh, PA 15213, USA

**Keywords:** post-traumatic hydrocephalus, traumatic brain injury, ventriculoperitoneal shunt, decompressive hemicraniectomy, shunt failure, cerebrospinal fluid

## Abstract

The development of hydrocephalus after severe traumatic brain injury (TBI) is an under-recognized healthcare phenomenon and can increase morbidity. The current study aims to characterize post-traumatic hydrocephalus (PTH) in a large cohort. Patients were prospectively enrolled age 16–80 years old with Glasgow Coma Scale (GCS) score ≤8. Demographics, GCS, Injury Severity Score (ISS), surgery, and cerebrospinal fluid (CSF) were analyzed. Outcomes were shunt failure and Glasgow Outcome Scale (GOS) at 6 and 12-months. Statistical significance was assessed at *p* < 0.05. In 402 patients, mean age was 38.0 ± 16.7 years and 315 (78.4%) were male. Forty (10.0%) patients developed PTH, with predominant injuries being subdural hemorrhage (36.4%) and diffuse axonal injury (36.4%). Decompressive hemicraniectomy (DHC) was associated with hydrocephalus (OR 3.62, 95% CI (1.62–8.07), *p* < 0.01). Eighteen (4.5%) patients had shunt failure and proximal obstruction was most common. Differences in baseline CSF cell count were associated with increased shunt failure. PTH was not associated with worse outcomes at 6 (*p* = 0.55) or 12 (*p* = 0.47) months. Hydrocephalus is a frequent sequela in 10.0% of patients, particularly after DHC. Shunt placement and revision procedures are common after severe TBI, within the first 4 months of injury and necessitates early recognition by the clinician.

## 1. Introduction

Post-traumatic hydrocephalus (PTH) is a serious but treatable sequelae of traumatic brain injury (TBI), with reports of its incidence ranging from 8% to 36% [1,2,3,4]. The pathophysiology of PTH remains unclear, but it is thought to be related to cerebrospinal fluid (CSF) outflow resistance and the disruption of drainage dynamics in the setting of neurotrauma [5]. Hydrocephalus is frequently described in patients who underwent decompressive hemicraniectomy (DHC) [5,6], however, it is also a complication in patients who did not undergo decompressive craniectomy for which additional relevant research is needed.

Delayed diagnosis and management of this phenomenon through ventricular shunting can delay rehabilitation and worsen long-term functional outcomes [7,8]. Prior findings in patient cohorts who underwent decompressive hemicraniectomy showed that younger age and the presence of interhemispheric hygroma were associated with PTH [1]. While low Glasgow Coma Scale (GOS) score, subarachnoid hemorrhage (SAH), and intraventricular hemorrhage (IVH) have been proposed by some to be associated with hydrocephalus [9,10], others did not find these factors to be predictive [1,11]. Thus, the development of hydrocephalus in the severe TBI population is further evaluated in the current study utilizing a longitudinal prospective cohort of patients with severe TBI managed at our institution. The measurable benefits of CSF diversion have been shown in 11–52% of patients on behavioral and functional tests [12,13]. On the other hand, shunt malfunction and complications arising from shunting procedures are well-known neurosurgical challenges that require repeated hospitalizations and revision operations. 

Intracranial hypertension is linked with poor prognosis after TBI [14,15], yet the potential impact of intracranial pressure (ICP), cerebral perfusion pressure (CPP) and brain tissue oxygenation (PbtO2) on the development of hydrocephalus has not been closely studied. To better characterize hydrocephalus after TBI, these clinical variables as well as demographic, radiographic, and CSF sample content are evaluated. This is followed by subgroup analysis of patients who underwent surgical decompression, developed shunt-hydrocephalus, and later on experienced shunt malfunction necessitating revision surgery. Therefore, the aims of this study are to characterize incidence of PTH in severe TBI patients with and without DHC, identify possible predictors of hydrocephalus, and investigate the outcomes of patients who experienced shunt malfunction in order to further guide physicians on clinical management. 

## 2. Materials and Methods

### 2.1. Study Design

All patients with severe TBI were prospectively enrolled in the Brain Trauma Research Center (BTRC) database at a single U.S. Level I trauma center from May 2000 to July 2014. Patients who met inclusion criteria and received treatment at the University of Pittsburgh Medical Center were (1) age of 16 and 80 years, (2) severe TBI defined as post-resuscitation Glasgow Coma Scale (GCS) score of ≤8, and (3) signed inform consent from health care proxies. The exclusion criteria of the study included imminent brain death (GCS score of 3 with fixed and dilated pupils), patients who were pregnant, and penetrating trauma. Neuromonitoring per standard of care was performed through the placement of external ventricular drain (EVD) and multi-modality intraparenchymal monitors (ICP, temperature, and PbtO2 probes). Elevated ICP was treated in a stepwise approach as outlined by the Brain Trauma Foundation Guidelines [16]. The study was approved by the local Institutional Review Board (IRB #PRO17030027) in accordance with the amended declaration of Helsinki.

### 2.2. Data Acquisition and Clinical Outcomes

Patient and clinical information (age, gender, admission GCS score, mechanism of injury), as well as hourly measurements of ICP, CPP, and PbtO2 were collected. Computed tomography (CT) imaging was reviewed to characterize the types of intracranial injury (epidural, subdural, intraparenchymal, ventricular). Diffuse axonal injury (DAI) was identified on magnetic resonance imaging (MRI) after initial stabilization from injury. Cerebrospinal fluid (CSF) diversion via shunting through placement of a ventriculoperitoneal or subdural-peritoneal shunt was performed. The clinical decision for ventriculoperitoneal shunt (VPS) placement included failure to wean off of EVD, decline in neurological exam, findings of progressive ventriculomegaly, increased extra-axial hygroma and elevated opening pressure (>20 cm H_2_O). CSF samples were collected by research assistants and stored at the biospecimens repository at the UPMC Department of Neurological Surgery. 

In patients with evidence of persistent elevated ICP that correlate with significant radiographic midline shift, and/or focal mass effect that were refractory to medical therapy, operative criteria were met and DHC was performed. Technical aspects of DHC can vary by institutional practice, however, in the present study, a unilateral frontotemporoparietal bone flap (12 × 15 cm) was performed, as previously described [4]. Outcomes from long term follow-up included variables pertaining to patients who experienced shunt malfunction. VPS failure was defined as the need for surgical revision. Glasgow Outcome Scale (GOS; 1 = death; 2 = persistent vegetative state; 3 = severe disability; 4 = moderate disability; 5 = low disability) was assessed at 6- and 12-months after trauma through structured follow-up evaluations by trained neuropsychologists at the BTRC.

### 2.3. Statistical Analysis

Descriptive statistics are presented as means, standard deviations (SDs) and standard errors (SEs) for continuous variables and proportions for categorical variables, interquartile range (IQR) depending on the data distribution. Categorical variables are presented using proportions. Differences in baseline characteristics between patients with and without post-traumatic hydrocephalus were assessed by Pearson’s chi-squared test (X^2^) and analysis of variance (ANOVA) for continuous variables. Fisher’s exact test was used to assess for differences in categorical variables when expected counts ≤5. For multivariable analysis, an inverse probability weighted logistic regression model was performed. Statistical significance was assessed at *p* = 0.05. All statistical analyses were conducted using a standard software package (Stata version 14.0, Stata Corp, College Station, TX, USA).

## 3. Results

**Figure 1:**
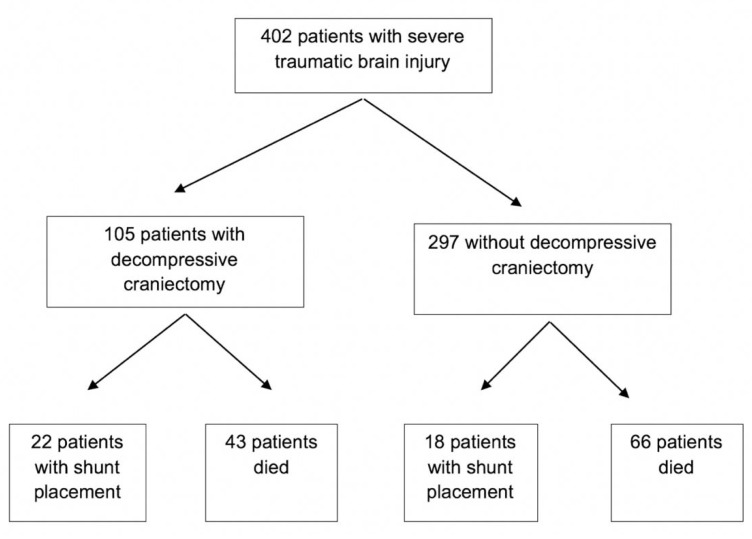
Flow chart for enrolled severe TBI patients who were enrolled in the study.

From May 2000 to July 2014, a total of 402 patients with severe blunt TBI were enrolled in the prospective study (Figure 1). The mean age was 38.0 ± 16.7 years old, and 78.4% of patients were male. Median (IQR) GCS score was 6 (1.0) on admission, median ISS was 30.0 (13.0), and median Marshall classification score on CT scan was 3.0 (4.0) (Table 1). The predominant types of intracranial hemorrhage were the following: subdural hematoma (SDH, 57.1%), intraparenchymal hemorrhage (IPH, 22.9%), epidural hematoma (EDH, 12.4%), and subarachnoid hemorrhage (SAH, 7.6%). DAI was identified in 16.2% of patients on MRI. Neuromonitoring showed that the initial ICP was 10.1 ± 8.6 mmHg, CPP was 78.9 ± 14.3 mmHg, and PbtO2 was 22.6 ± 14.4 mmHg in our cohort of patients. 

The univariate analysis demonstrated that gender, GCS score, intracranial bleeding pattern, and the presence of DAI were not significantly associated with developing hydrocephalus (Table 1). There were less patients with SAH who needed VPS placement compared to those who did not need a shunt (7.5% versus 19.3%, *p* = 0.04). There were no differences in ICP recording or PbtO2 between patients with and without hydrocephalus. Decompressive craniectomy was associated with the development of hydrocephalus. There were 297 (73.9%) patients who did not undergo decompressive hemicraniectomy, compared to 105 (26.1%) patients who needed decompression for cerebral edema and/or refractory intracranial hypertension. Eighteen (6.1%) patients who did not need DHC developed hydrocephalus, while 22 (21.0%) patients who underwent DHC developed hydrocephalus and needed VPS placement (*p* < 0.01).

Multivariate logistic regression with inverse probability weight analysis to adjust for mortality showed that patients undergoing a DHC was the only significant predictor of need for CSF diversion (Table 2). While age was not associated with overall risk of PTH in the current study, subgroup analysis found that compared to patients without hydrocephalus after DHC, those who developed hydrocephalus after DHC were younger (35.5 ± 17.7 versus 46.0 ± 17.7 years old, *p* < 0.01). In addition, patients who developed hydrocephalus after DHC had higher indexes of polytrauma (median ISS 35 versus 26, *p* = 0.04). Compared to those without DHC, patients who had DHC were associated with increased odds of VPS placement in the future (unadjusted OR 3.75, 95%CI (1.92–7.31), *p* < 0.01; adjusted OR 3.62, 95%CI (1.62–8.07), *p* < 0.01). Patients who developed post-traumatic hydrocephalus following decompressive craniectomy achieved equivalent neurologic outcomes compared to those who did not develop PTH (6-month GOS of 3 IQR [1] versus GOS 1 IQR [2], *p* = 0.18; 12-month GOS of 3 IQR [2] versus 1 IQR [3], *p* = 0.24). Table 3 illustrates that the differences in baseline CSF cell count, from sampling prior to shunt placement, were not associated with increased risk of shunt failure requiring revision surgery in the future.

## 4. Discussion

Our institutional experience from 2000 to 2014 of a prospective cohort of 402 patients demonstrates that the overall rate of hydrocephalus following severe TBI is 10.0%. The risk of developing hydrocephalus was notably greater in patients who required DHC at a rate of 21.0%, and for this reason, DHC was the strongest predictor of post-traumatic hydrocephalus. PTH is an important sequela that can develop in TBI patients, and if untreated results in increased morbidity, mortality, and healthcare burden [17,18]. Landmark randomized trials in the past decade, including the DECRA and RESCUEicp investigations [19,20], showed that DHC reduces intracranial hypertension, mortality, and outcomes of severe disability in severe TBI patients at 6 months, but its use necessitates understanding of the associated postsurgical risks [21].

The pathophysiology for hydrocephalus after decompression remains unclear, which may be due to impaired CSF pulsatility, increased CPP, disruption of drainage pathways, and inflammation of arachnoid granulations [5,22]. Findings from prior studies have been equivocal on possible factors that could be associated with hydrocephalus, including low GCS score, presence of SAH and IVH, interhemispheric hygromas, and younger age [1,9,23]. Recently, it has been shown that genetic factors could also play a role in ICP variability after TBI [24,25]. To minimize risk of interhemispheric subdural hygromas, Bonis et al. recommended performing the craniectomy with the superior margin at least 2.5 cm lateral to the midline [26]. In the present study, we examined a spectrum of demographic and clinical characteristics for possible risk factors of hydrocephalus utilizing a large prospective severe TBI cohort, and we found that the most important predictor for hydrocephalus was the need for DHC.

Surgical decompression may be a life-saving procedure, but it is not without secondary risks [27,28]. The common surgical morbidities associated with decompressive craniectomy and also with the subsequent cranioplasty include cortical herniation, subdural effusion, hydrocephalus, seizure, and infection. The etiology for hydrocephalus after DHC is likely multifactorial and not fully understood. Experimental models have found that after craniectomy, the effects of the skull and dura on CSF hydrodynamics are altered to induce a two-fold increase in CSF outflow resistance, as well as greater brain compliance as measured by the pressure-volume index [29,30]. In the present study, while the factor of age did not play a role in the non-DHC cohort, notably, we demonstrate that younger patients who had DHC were more likely to develop shunt-dependent hydrocephalus. This is likely because the cerebral physiology in younger patients, i.e., greater cerebral volume and compliance in contrast to older adults with cerebral atrophy, are more prone to perturbations to CSF circulation [1].

Ventriculoperitoneal shunt procedures are common neurosurgical procedures with reported overall rate of shunt failure ranging from 15% to 32% [31,32,33]. The prevalence of intracranial hemorrhage and wound contamination in TBI makes neurotrauma patients at greater risk for shunt failure and requiring revision surgery. In our patients, the rate of shunt failure was 45.0%, with median insertion-to-failure time of 4 months. We did not identify any predictors for shunt failure despite increased need for shunt revision surgery in the TBI population, including the primary type of hemorrhage, time to shunt insertion or the use of a programmable valve. While programmable valve designs may be able reduce the number of revisions in specific sub-population of patients with normal pressure hydrocephalus [34], programmable valves, placed in 75% of our patients, did not reduce the incidence of shunt failure requiring revision.

Lastly, high CSF protein concentration or RBC level has been postulated as a concern for shunt failure due to increased viscosity and obstruction risk. The pediatric literature is equivocal regarding the association between high CSF cell concentration, viscosity and shunt complications [35,36,37]. Similarly, in a predominantly adult severe TBI patient population, we find that differences in CSF cell concentration are not predictors of future shunt failure. Thus, shunt placement should not be delayed and be performed at the earliest clinical indication in order to facilitate neurological recovery.

### Limitations

This study is not without limitations. The data are analyzed retrospectively from a prospectively collected cohort of patients treated over fourteen years. As such, there is inherent variability in medical management protocols and in surgical techniques between surgeons with respect to the size of the craniectomy and approach to expansile duroplasty. The sample size of VPS shunt failure over the years has been small. Because our 2-year follow-up rate in our severe TBI database is over 80%, we do not believe that the risk of missing data meaningfully impacts our analysis. The impact of choice of shunt design and cranioplasties performed also could not be further elucidated. In spite of this, results from the present investigation add to the existing knowledge on hydrocephalus in the severe TBI population and shunt failure at follow-up necessitating reoperation surgery.

## 5. Conclusions

Shunt-dependent hydrocephalus is a serious complication after neurotrauma, with an overall incidence of 10.0% in severe TBI patients. Hydrocephalus commonly occurs in those who underwent decompressive hemicraniectomy at 21.0% and with younger patients. Timely diagnosis of PTH and appropriate shunt surgery can help patients achieve similar functional outcomes relative to their counterparts who did not have PTH.

## Figures and Tables

**Table 1:** Baseline demographics and clinical characteristics of severe TBI patients.

Parameters	Total (*n* = 402)	No Shunt (*n* = 362)	Shunt for PTH (*n* = 40)	*p* Value
Age, years (mean, SD)	38.0 (16.7)	38.4 (16.8)	34.5 (16.2)	0.16
Male gender, %	78.4	77.9	82.5	0.69
Marshall score, median (IQR)	3.0 (4.0)	3.0 (4.0)	3.0 (1.0)	0.31
ISS, median (IQR)	30.0 (13.0)	30.0 (13.0)	35.0 (12.0)	0.32
GCS, median (IQR)	6.0 (1.0)	6.0 (2.0)	6.0 (2.0)	0.29
Primary intracranial bleed, %				
EDH	7.2	6.9	10.0	0.32
SDH	32.6	31.8	40.0	0.19
SAH	18.2	19.3	7.5	**0.04**
ICH	31.3	30.9	35.0	0.36
IVH	2.7	2.8	2.5	1.00
Diffuse axonal injury, %	36.4	35.7	42.3	0.33
Neurosurgical intervention, %				
EVD	58.5	57.7	65.0	0.24
DHC	27.6	24.6	55.0	**<0.01**
Neuromonitoring, mean (SD)				
ICP, mmHg	10.1 (8.6)	9.9 (7.9)	10.1 (8.8)	0.90
PbtO2, mmHg	22.6 (14.4)	22.8 (14.4)	21.6 (14.7)	0.69
CPP, mmHg	78.9 (14.3)	78.6 (14.1)	80.7 (15.4)	0.51

TBI = traumatic brain injury; PTH = posttraumatic hydrocephalus; IQR = interquartile range; ISS = injury severity score; GCS = Glasgow coma scale; EDH = epidural hematoma; SDH = subdural hematoma; SAH = subarachnoid hemorrhage; ICH = intracerebral hemorrhage; IVH = intraventricular hemorrhage; EVD = external ventricular drain; DHC = decompressive hemicraniectomy; SD = standard deviation; ICP = intracranial pressure; PbtO2 = brain tissue oxygenation; CPP = cerebral perfusion pressure. Bold is used for significance *p* = 0.05.

**Table 2:** Severe TBI patients who developed shunt-dependent hydrocephalus.

Parameters	Total with Shunt (*n* = 40)	Shunt Failure (*n* = 18)	No Shunt Failure (*n* = 22)	*p* Value
Age, years mean (SD)	34.5 (16.2)	29.7 (23.2)	36.9 (14.4)	0.14
Male gender, %	82.5	83.3	87.5	0.56
ISS, median (IQR)	35.0 (12.0)	30.0 (10.0)	38.0 (15.0)	0.06
GCS, median (IQR)	6.0 (2.0)	6.0 (1.0)	6.5 (2.0)	0.26
Trauma to shunt, days median (IQR)	35.0 (53.5)	32.0 (16.0)	33.5 (1163)	0.29
Follow up, days median (IQR)	1814 (2803)	1871 (2047)	1819 (3510)	0.82
Primary bleed, %				0.17
EDH	10.0	22.2	0.0	
SDH	40.0	33.3	50.0	
SAH	7.5	11.1	0.0	
ICH	35.0	27.8	43.8	
Diffuse axonal injury, %	42.3	54.5	30.0	0.25
Programmable valve, %	75.0	88.9	68.8	0.15
GOS, median (IQR)				
6 months	3.0 (1.5)	3.0 (1.0)	3.0 (0.5)	0.55
12 months	3.0 (2.0)	3.0 (1.0)	3.0 (1.5)	0.47

TBI = traumatic brain injury; PTH = posttraumatic hydrocephalus; SD = standard deviation; ISS = injury severity score; IQR = interquartile range; GCS = Glasgow coma scale; EDH = epidural hematoma; SDH = subdural hematoma; SAH = subarachnoid hemorrhage; ICH = intracerebral hemorrhage; IVH = intraventricular hemorrhage; EVD = external ventricular drain; DHC = decompressive hemicraniectomy; SD = standard deviation; GOS = Glasgow outcome score.

**Table 3:** CSF content prior to shunt placement in patients with PTH.

CSF Content	Shunt Failure	No Shunt Failure	*p* Value
RBC, cells/mm^3^	2376 ± 9312	840.9 ± 1883	0.48
WBC, cells/mm^3^	3.9 ± 11.1	1.7 ± 1.6	0.40
Glucose, mg/dL	66.5 ± 9.9	62.1 ± 19.7	0.41
Protein, mg/dL	57.4 ± 83.3	51.0 ± 38.28	0.76

CSF = cerebrospinal fluid; PTH = posttraumatic hydrocephalus; RBC = red blood cell; WBC = white blood cell.

## Data Availability

The datasets generated during and/or analyzed during the current study are not publicly available but could be acquired from the corresponding author on reasonable request.
